# Development and Exploitation of KASP Assays for Genes Underpinning Drought Tolerance Among Wheat Cultivars From Pakistan

**DOI:** 10.3389/fgene.2021.684702

**Published:** 2021-06-09

**Authors:** Shoaib Ur Rehman, Muhammad Ali Sher, Muhammad Abu Bakar Saddique, Zulfiqar Ali, Mahmood Alam Khan, Xinguo Mao, Ahsan Irshad, Muhammad Sajjad, Rao Muhammad Ikram, Mahnoor Naeem, Ruilian Jing

**Affiliations:** ^1^Institiute of Plant Breeding and Biotechnology, Muhammad Nawaz Shareef University of Agriculture, Multan, Pakistan; ^2^National Key Facility for Crop Gene Resources and Genetic Improvement, Institute of Crop Sciences, Chinese Academy of Agricultural Sciences, Beijing, China; ^3^National Engineering Laboratory of Crop Molecular Breeding, National Center of Space Mutagenesis for Crop Improvement, Institute of Crop Sciences, Chinese Academy of Agricultural Sciences, Beijing, China; ^4^Department of Biosciences, COMSATS University Islamabad (CUI), Islamabad, Pakistan; ^5^Department of Agronomy, Muhammad Nawaz Shareef University of Agriculture, Multan, Pakistan

**Keywords:** gel-free markers, KASP markers, drought related genes, Pakistani wheat, genetic diversity

## Abstract

High-throughput genotyping for functional markers offers an excellent opportunity to effectively practice marker-assisted selection (MAS) while breeding cultivars. We developed kompetitive allele-specific PCR (KASP) assays for genes conferring drought tolerance in common wheat (*Triticum aestivum* L.). In total, 11 KASP assays developed in this study and five already reported assays were used for their application in wheat breeding. We investigated alleles at 16 loci associated with drought tolerance among 153 Pakistani hexaploid wheat cultivars released during 1953–2016; 28 diploid wheat accessions (16 for AA and 12 for BB) and 19 tetraploid wheat (AABB) were used to study the evolutionary history of the studied genes. Superior allelic variations of the studied genes were significantly associated with higher grain yield. Favored haplotypes of *TaSnRK2.3*-1A, *TaSnRK2.3*-1B, *TaSnRK2.9*-5A, *TaSAP*-7B, and *TaLTPs*-1A predominated in Pakistani wheat germplasm indicating unconscious pyramiding and selection pressure on favorable haplotypes during selection breeding. *TaSnRK2.8*-5A, *TaDreb*-B1, *1-feh w3*, *TaPPH*-7A, *TaMOC*-7A, and *TaPARG*-2A had moderate to low frequencies of favorable haplotype among Pakistani wheat germplasm pointing toward introgression of favorable haplotypes by deploying functional markers in marker-assisted breeding. The KASP assays were compared with gel-based markers for reliability and phenotypically validated among 62 Pakistani wheat cultivars. Association analyses showed that the favorable allelic variations were significantly associated with grain yield-contributing traits. The developed molecular marker toolkit of the genes can be instrumental for the wheat breeding in Pakistan.

## Introduction

Crop improvement strategies have always circumambulated yield-enhancing genes. Therefore, exploitation of gene diversity in breeding germplasm and identification of superior genetic variations are prioritized activities in crop genetic improvement. Such exploitations enable breeders to identify desirable germplasm for breeding and to devise strategies for pyramiding superior genetic variations for targeted traits. Wheat is one of the most important cereal crops, and there is demand for a yield increase of up to 50% by 2050 (Curtis and Halford, 2014). Being a staple food crop in Pakistan, wheat growth and development is severely influenced by abiotic stress, resulting in a significant reduction in grain yield. Moreover, the genetic structure of modern Pakistani wheat cultivars built around only a few cultivars such as Bluebird, Kauz, Kalyansona, and Buho, and there is an urgent need to introduce new diversity for sustainable wheat production in Pakistan. Therefore, the utilization of genes conferring drought tolerance is regarded as an effective way to ensure high and sustainable yield in wheat. Marker-assisted selection (MAS) based on pyramiding superior alleles/haplotypes is considered as a potential strategy to wheat improvement for economically important traits. The challenge is to deploy such strategy in breeding programs in a time- and cost-efficient manner for different scenarios (Richards et al., 2014).

*TaSnRK2*.*3*-1A/1B (Miao et al., 2017), *TaSnRK2*.*9*-5A (Ur Rehman et al., 2019), *TaPARG*-2A (Li B. et al., 2016), *TaSAP*-7B (Wang et al., 2018), *TaPPH*-7A (Wang et al., 2019), and *TaMOC1*-7A (Zhang B. et al., 2015) are associated with higher grain yield under water stress conditions. *TaSnRK2*.*8*-5A associated with higher seedling biomass under normal conditions and water-soluble carbohydrates under limited irrigation conditions (Zhang et al., 2013), *TaLTPs*-1A associated with ideal plant height under drought conditions (Li Q. et al., 2016), and *TaDreb*-B1 (Wei et al., 2009) and *1*-*feh w3* (Zhang J. et al., 2015) are also reported as drought tolerance-conferring genes.

Functional markers (FMs) of the aforementioned genes were successfully applied in Chinese wheat cultivars and provided the concept of screening of genotypes for wheat breeding in Pakistan. Various single-marker methods have been developed for single-nucleotide polymorphism (SNP) genotyping, such as cleaved amplified polymorphic sequences (CAPS), and derived cleaved amplified polymorphic sequences (dCAPS). The CAPS and dCAPS markers are relatively low throughput, laborious, and cost ineffective, as they rely upon site-specific cleavage of PCR products with restriction enzymes and require gel electrophoresis to separate products. At present, more than 150 FMs are available for important genes, giving plant breeders a molecular toolkit for the selection of favorable traits (Liu et al., 2012). Although FMs are available for wheat, their deployment retains limited courtesy cost and time needed to exploit larger populations.

Kompetitive allele-specific PCR (KASP) is a uniplex and flexible genotyping platform which achieves high throughput in a time- and cost-effective way (Semagn et al., 2014). Conversion of conventional FMs into KASP assays could greatly speed up improvement in breeding programs. Therefore, the aims of the present study are (i) to develop KASP-based assays of FMs for higher grain yield and drought-conferring genes in wheat, (ii) to perform marker trait association analyses among Pakistani wheat cultivars and to investigate the distribution of FMs in wheat cultivars across Pakistan, and (iii) to know the genetic diversity of given genes among diploid, tetraploid, and hexaploid wheat. The information will be useful in breeding wheat for higher grain yield and drought tolerance by MAS.

## Materials and Methods

### Germplasm

One hundred and fifty-three wheat cultivars from Pakistan released during 1953–2016 were used to identify favorable haplotype frequencies of drought tolerance-conferring genes (Supplementary Table 1). The wheat collection comprised four groups based on time of release, i.e., pre-green revolution 1953–1965, post-green revolution 1966–1985, 1986–2005, and post-2005. Besides *Triticum aestivum* germplasm, nine genotypes of *T. urartu* (AA), four genotypes of *T. boeoticum Bioss* (AA), three genotypes of *T. monococcum* (AA), 12 genotypes *Aegilops speltoides* (BB), five genotypes of *T. dicoccum* L. (AABB), three genotypes of *T. persicum* Vav (AABB), five genotypes of *T. dicoccoides* Koern (AABB), four genotypes of *T. polonicum* L., and two genotypes of *T. turgidum* (AABB) (Supplementary Table 2) were also used to identify the polymorphic information contents (PIC) and gene diversity of the studied genes. Wheat genotypes such as Chinese Spring, MexiPak-65, and Parwaz-94 were used as controls for the identification of particular alleles. A subset of 62 wheat genotypes (55 modern cultivars and seven landraces) from 153 wheat cultivars were also grown at MNS University of Agriculture, Multan and Quaid-e-Azam University, Islamabad, under two water regimes, i.e., under water stress at flowering stage and under normal conditions. Randomized complete block design was followed with duplicates at both locations. Standard agronomic practices were followed to ensure proper plant stand. Wheat sown under normal conditions was irrigated initially after 25 days of sowing followed by irrigation at flowering and grain filling stages. The wheat grown under the water stress regime was irrigated only once after 25 days of sowing. Precipitation mainly occurred at the end of March at both locations. Water contents of different soil profiles are given in Supplementary Table 3. Each experimental plot was 6 m in length with six rows having a row spacing of 30 cm with ∼40 seeds per row. The cultivars were sown in mid-November 2019 and harvested in April of the following year. These 62 genotypes were planted for traits, i.e., plant height (PH), thousand kernel weight (TKW), and grains per spike (GPS), under both water regimes. Association analysis was performed on the average of all the parameters from both water regimes, and a phenotypic comparison of allelic variations was presented.

### Genotyping

**FIGURE 1:**
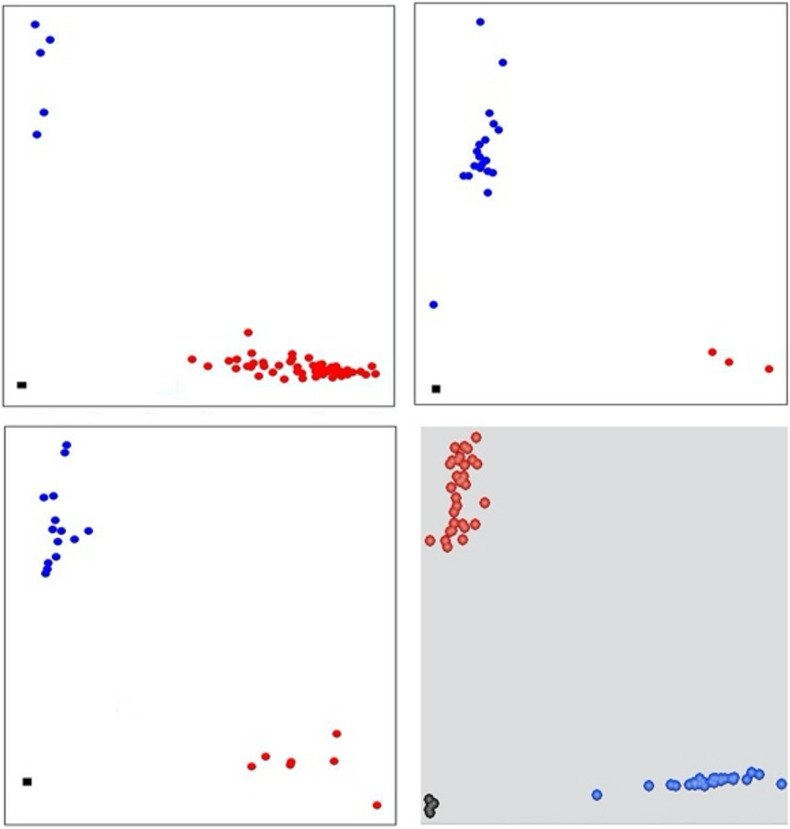
Scatter plot for selected KASP assays showing clustering of genotypes on the *Y*- and *X-*axes. Genotypes colored red have a HEX-type allele; genotypes colored blue have a FAM-type allele; black dots represent non-template control. Top left and right corners, KASP assays for *TaSnRK2.3*-1A and *TaSnRK2.3*-1B, respectively. Bottom left and right corner, KASP assays for *TaSAP*-7B and *TaLTPs*, respectively.

Five KASP assays were selected from published reports including two SNPs for *TaSnRK2*.*9*-5A (Ur Rehman et al., 2019) and one each for *TaMOC1*-7A, *TaDreb*-1B, and *1*-*feh w3* (Rasheed et al., 2016). The remaining 11 KASP assays were developed in this study (Figure 1). The information on the selected genes, the sequence polymorphism, KASP assays, and their sources are provided in Supplementary Table 4.

For the KASP assays developed in this study, the nucleotide sequences of drought tolerance-causing genes were retrieved from the published literature. The diagnostic polymorphic sites were identified, and KASP primers were developed following standard KASP guidelines. The allele-specific primers were designed carrying the standard FAM and HEX tails and with the targeted SNP at the 3′ end. A common primer was designed so that the total amplicon length was less than 120 bp. The primer mixture comprised 46 μl ddH_2_O, 30 μl common primer (100 μM), and 12 μl of each tailed primer (100 μM). Assays were tested in 96-well formats and set up as 5 μl reaction mixture (2.4 μl of 25 ng/μl DNA, 2.4 μl of 2 × KASP master mixture, 0.06 μl of primer mixture, 0.04 MgCl_2_, and 0.1 μl of ddH_2_O). PCR cycling was performed using the following protocol: hot start at 95°C for 15 min, followed by 10 touchdown cycles (95°C for 20 s; touchdown at 65°C initially and decreasing by −1°C per cycle for 25 s), followed by 32–35 additional cycles of annealing (95°C for 15 s, 57°C for 1 min). Fluorescence levels were detected and analyzed by using CFX Connect Real-Time PCR detection system (Bio-Rad^®^ laboratories Inc. United States) and QuantStudio 7 Flex Real-Time PCR systems.

### Statistical Analyses

Student’s *t*-test at *P* < 0.05 was used to check the effect of SNP/haplotype on the studied agronomic traits. Allele/haplotype frequencies were calculated for all loci. PIC and gene diversity (*H*_*e*_) were calculated for each locus using https://www.gene-calc.pl/pic.

## Results

### Comparison of KASP Markers and Conventional Gel-Based PCR Markers

The results from KASP markers were compared to contrasting gel-based markers for all the genes. All studied KASP assays produced consistent results when compared to conventional PCR markers in 23 diverse wheat genotypes (Supplementary Table 5), but for *TaPARG*-2A-KASP-10 (C/T), the concentration of each tailed primer increased up to 15 μM to obtain satisfactory results.

### KASP Assays for Grain Yield-Contributing Traits

**FIGURE 2:**
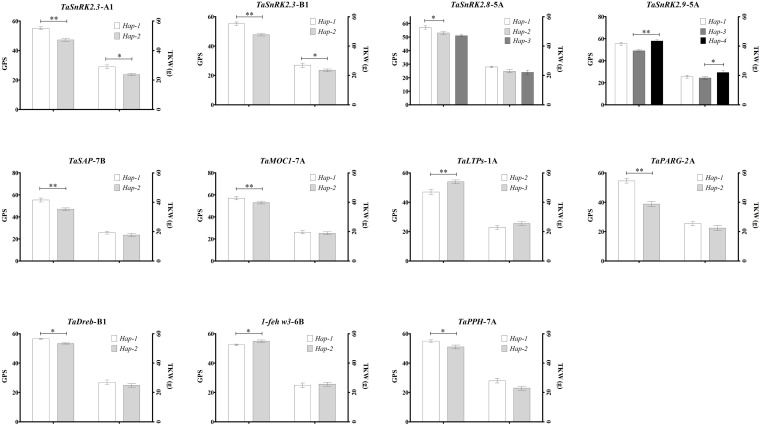
Phenotypic comparison of allelic variations. Traits are thousand kernel weight—TKW, grains per spike—GPS, and plant height—PH. ^∗^*P* < 0.05, ^∗∗^*P* < 0.01. Error bar denotes standard deviation.

Association analyses of allelic variations of the studied genes showed that the favorable allelic variations were significantly associated with higher grain yield traits among the studied 62 Pakistani wheat germplasm (Figure 2). Haplotypes associated with grain-related traits are *Hap*-1 (CA) of *TaSnRK2*.*3*-1A and *Hap*-1 (CG) of *TaSnRK2*.*3*-1B which are favored for higher GPS and TKW (Figure 2). *Hap*-1/3 (A-A/G allele accessions) of *TaSnRK2*.*8*-5A showed a non-significant association with higher GPS and TKW. *Hap*-1 (TA) and *Hap*-4 (CA) of *TaSnRK2*.*9*-5A are also associated with higher GPS and TKW (Figure 2). For *TaSAP*-7B, accessions carrying the “C” allele possess ideal plant height (99 cm) and higher GPS and TKW. Accessions carrying *Hap*-H of *TaMOC1*-7A possess higher GPS and TKW than *Hap*-L. *Hap*-3 (*GC*) of *TaLTPs* associated with ideal plant height (100 cm) and higher GPS and TKW. *TaDreb*-B1 and *1*-*feh w3* accessions carrying the *Hap-*1 “A-allele” and *Hap-*1 “C-allele,” respectively, are associated with higher grain yield contributing parameters (Figure 2). *Hap*-1 (CC) of *TaPARG*-2A showed an association with higher GPS. Favorable allelic variation of *TaPPH*-7A-1 (“A” allele) also showed an association with higher GPS among Pakistani wheat cultivars (Figure 2).

In general, frequencies of favored haplotypes and/or alleles were higher in 153 Pakistani wheat germplasm released from 1953 to 2016. For *TaSnRK2*.*3*-1A, 127 (83.01%) Pakistani wheat cultivars had the desirable haplotype (*Hap*-1). The favorable haplotype (*Hap*-1) of *TaSnRK2*.*3*-1B was also present in 127 (83.01%) Pakistani wheat cultivars (Table 1). For *TaSnRK2*.*8*-5A, the frequency of preferred allele “A” was low (33.99%) in given wheat cultivars. The frequency of favored haplotypes for *TaSnRK2*.*9*-5A (*Hap*-1 and *Hap*-4) was 75.82% and 2.61%, respectively. The favored allele for *TaSAP*-7B was predominant in 129 (84.31%) wheat cultivars; at *TaMOC1*-7A, 33 (21.57%) desirable haplotypes (*Hap*-H). The favorable haplotype (*Hap*-3) of *TaLTPs* was present in 146 (95.42%) wheat cultivars. Superior alleles for *TaDreb*-B1, *1*-*feh w3*, and *TaPPH-7A* were present in 48 (31.37%), 44 (28.75%), and 67 (43.79%) wheat cultivars. The favorable haplotypes of *TaPARG*-2A (*Hap*-2 (1.31%) and *Hap*-3 absent) were present in very low frequencies among given wheat germplasm (Table 1).

**TABLE 1:** Allelic frequencies in 153 Pakistani wheat cultivars.

Gene	Locus	Haplotype	Genotype	Number of accession	Phenotype	Frequency (%)	Reference (of phenotype)
*TaSnRK2.3*	*TaSnRK2.3*-1A	*Hap*-1	CA	127	Higher TKW	83.01	Miao et al., 2017
		*Hap*-2	TG	24		15.69	
		*Hap*-3	CG	2		1.31	
	*TaSnRK2.3-*1B	*Hap*-1	CG	127	Higher TKW	83.01	
		*Hap*-2	TC	7		4.58	
		*Hap*-3	CC	19		12.42	
*TaSnRK2.8*	*TaSnRK2.8-*5A	*Hap*-1	A	52	Seedling biomass and water-soluble carbohydrates	33.99	Zhang et al., 2013
		*Hap*-2	G	69		45.10	
			A/G	32		20.92	
*TaSnRK2.9*	*TaSnRK2.9-*5A	*Hap*-1	TA	116	Higher TKW	75.82	Ur Rehman et al., 2019
		*Hap*-2	TC	2		1.31	
		*Hap*-3	CC	31		20.26	
		*Hap*-4	CA	4	Higher GPS	2.61	
*TaSAP*	*TaSAP-*7B	*Hap*-1	C	129	Higher TKW and short PH	84.31	Wang et al., 2018
		*Hap*-2	T	24		15.69	
*TaMOC*	*TaMOC1-*7A	*Hap*-H	G	33	Higher grain number	21.57	Zhang B. et al., 2015
		*Hap*-L	A	120	Lower grain number	78.43	
*TaLTPs*	*TaLTPs-*1A	*Hap*-1	AC	0		0.00	Li Q. et al., 2016
		*Hap*-2	GT	7		4.58	
		*Hap*-3	GC	146	Ideal plant height	95.42	
*TaPARG*	*TaPARG-*2A	*Hap*-1	CC	151		98.69	Li B. et al., 2016
		*Hap*-2	TC	2	Lower PH, ETN, and higher TKW	1.31	
		*Hap*-3	TT	0		0.00	
*TaDreb*	*TaDreb-*B1	*Hap*-1	A	48	Drought tolerance	31.37	Wei et al., 2009
		*Hap*-2	C	105		68.63	
1-FEH W3	*1-feh w3-*6B	*Hap*-1	C	44	Drought tolerance	28.76	Zhang J. et al., 2015
		*Hap*-2	T	109		71.24	
*TaPPH*	*TaPPH-*7A	*Hap*-1	A	67	Higher TKW and short PH	43.79	Wang et al., 2019
		*Hap*-2	G	86		56.21	

Certain combinations of two or more desirable alleles or haplotypes tended to occur in higher frequencies in one group more than the other; for example, TaSnRK2.3-1A + TaSnRK2.3-1B + TaSnRK2.9-5A + TaSAP-7B + TaLTPs-1A were present in 110 (71.89%) wheat cultivars.

### Selection Frequencies of Favored Alleles/Haplotypes in Pakistan Since 1953

**FIGURE 3:**
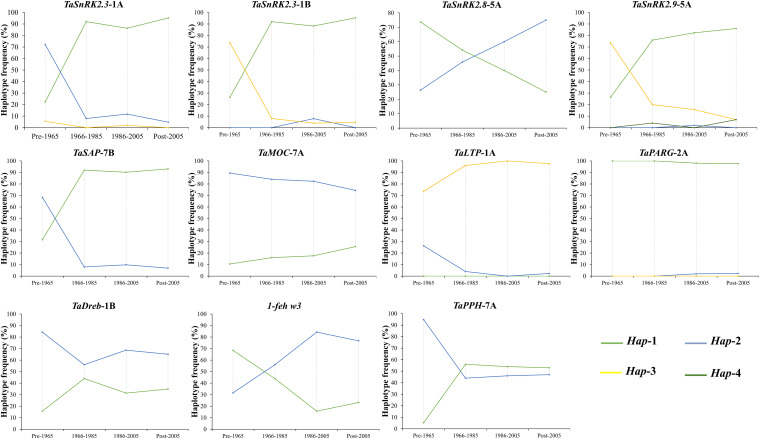
The frequency of drought-tolerant genes’ haplotypes in Pakistani wheat cultivars released in different eras.

Since 1953, the frequency distribution of favored haplotypes of given drought tolerance-responsible genes varied among Pakistani wheat germplasm (Figure 3). For *TaSnRK2*.*3*-1A/1B, 83.01% wheat accessions contained favored haplotypes. For *TaSnRK2*.*9*-5A, *TaLTPs*-1A, and *TaSAP*-7B, 78.41%, 95.42, and 84.31% Pakistani wheat accessions possessed favorable allelic variations, respectively. Based on released time, Pakistani wheat cultivars were divided into four groups. From 1953 to 2016, the frequency of favored haplotypes for *TaSnRK2*.*3*-1A, *TaSnRK2*.*3*-1B, *TaSnRK2*.*9*-5A, *TaSAP*-7B, and *TaLTPs*-1A increased remarkably. The combined frequencies of favored alleles/haplotypes of aforementioned genes increased from 35.78% in the pre-1965s to 93% in the post-2005s, showing a progressive selection of favored alleles/haplotypes over the years. The frequencies of favored alleles/haplotypes of other drought-conferring genes remain low (<22%) since 1953, suggesting the potential of favored allele/haplotype introgression through FMs developed in this study.

### Diversity Pattern in Wheat Germplasm

To survey the evolutionary history of given drought tolerance-responsible genes, we analyzed the given genes in wheat progenitor accessions. The results showed that during polyploidization events, diversity decreased in a given set of genes. Diploid wheat accessions showed an average of 0.265 (PIC) and 0.0.331 (H_*e*_) in the investigated genes. The studied tetraploid (AABB) accessions showed an average 0.361 (PIC) and 0.418 (H_*e*_) in the given genes. Hexaploid Pakistani wheat showed an average 0.270 (PIC) and 0.317 (H_*e*_) in the given genes (Table 2). The results depict that the overall diversity for the given genes (except *TaSnRK2.8*-5A, *TaDreb*-B1, and *TaPPH*-7A) among Pakistani wheat accession reduced.

**TABLE 2:** Polymorphic information contents and gene diversity in diploid, tetraploid, and hexaploid Pakistani wheat.

Genes	Diploid wheat			Tetraploid wheat			Hexaploid wheat		
	Alleles/Haplotypes	PIC	*H*_e_	Alleles/Haplotypes	PIC	*H*_e_	Alleles/Haplotypes	PIC	*H*_e_
*TaSnRK2.3-*1A	2	0.304	0.375	3	0.414	0.498	3	0.250	0.285
*TaSnRK2.3-*1B				3	0.504	0.570	3	0.270	0.294
*TaSnRK2.8-*5A	2	0.370	0.490	2	0.357	0.466	3	0.562	0.637
*TaSnRK2.9-*5A				3	0.512	0.575	4	0.334	0.381
*TaSAP-*7B				2	0.276	0.331	2	0.232	0.268
*TaMOC1-*7A				2	0.368	0.487	2	0.284	0.343
*TaLTPs-*1A				2	0.276	0.331	3	0.090	0.095
*TaPARG-*2A	2	0.121	0.130	2	0.487	0.368	3	0.019	0.019
*TaDreb-*B1				2	0.090	0.09	2	0.335	0.427
*1-feh w3*				2	0.374	0.498	2	0.327	0.411
*TaPPH*-7A				2	0.310	0.384	2	0.371	0.492
Sum		0.796	0.995	25	3.973	4.607	29	2.707	3.165
Average	2	0.265	0.331	2.27	0.361	0.418	2.64	0.270	0.317

## Discussion

Novel genomic tools provide an opportunity in meeting the challenge of enhanced genetic gain to safeguard sustainable production. The application of molecular markers to accelerate MAS has proven successful in wheat breeding programs (Rasheed et al., 2016). Moreover, the concepts of MAS in wheat are now transformed into genomic selection methods to improve genetic gains (Zhao et al., 2019). It has been reported that the use of functional markers for individual genes can significantly improve prediction accuracies (Rutkoski et al., 2014). Using functional markers and genes for wheat breeding, the appropriate breeding material should be selected based on production needs (Hao et al., 2020). Breeder friendliness, high throughput, and cost-effectiveness are the main considerations in selecting an appropriate genotyping platform for genomic selection and MAS (Semagn et al., 2014). Here we have demonstrated the effectiveness of newly developed KASP assays for genes conferring drought tolerance in wheat. These assays offer fast-track ways to deploy drought tolerance-causing genes in wheat improvement in a cost-effective manner.

### Reliability of Developed KASP Assays

Identification and validation of SNPs is a significant challenge in wheat due to the large genome size, polyploidy, and high percentage of repetitive sequences (Ramirez-Gonzalez et al., 2015). Hence, it is necessary to validate the SNPs. The developed KASP assays were validated for reliability. The KASP assays were compared to their equivalent gel-based markers on a small but diverse set of Chinese wheat germplasm and check cultivars with known alleles at each gene. One KASP assay (*TaSnRK2*.*8*-5A-KASP-7) showed inconsistent outcomes in the form of heterozygous conditions for the given alleles. Overall, the conversion rate for newly developed KASP assays was > 98% and were able to convert gel-based PCR markers into breeder friendly gel-free KASP markers.

### Allelic Variation at Loci Influencing Grain Related Traits

MAS of superior alleles in breeding programs is important for the ongoing improvement of wheat. The deployment of superior alleles in improved cultivars could be enhanced if efficient molecular diagnostics are available (Rasheed et al., 2017). TKW, GPS, and PH are important yield-contributing traits in wheat, and recently several genes affecting these traits were cloned. Favorable allelic variations of the genes studied in this work have been reported to be associated with higher grain weight and higher grain number under normal and water stress conditions in Chinese wheat germplasm (Table 1). The investigation of Pakistani wheat germplasm for these genes is necessary for assessing the effect of selection pressure on favorable haplotypes and to alert wheat breeders for these favorable variations for grain yield. Our results suggested strong selection pressure on favorable haplotypes at *TaSnRK2*.*3*-1A/1B, *TaSnRK2*.*9*-5A, *TaSAP*-7B, and *TaLTPs*-1A among Pakistani wheat accessions. A moderate frequency of favored haplotypes was observed at *TaSnRK2*.*8*-5A, *TaDreb*-B1, *TaPPH*-7A, and *1-feh w3*, indicating that exploitation of these alleles may be continued to gain a yield increase in Pakistan. This unconscious selection of favorable haplotypes is likely due to the high linkage disequilibrium of important genes selected during selection breeding. The given Pakistani wheat germplasm had high frequencies of unfavorable allelic variations for *TaMOC*-7A and *TaPARG*-2A, suggesting the potential of favorable haplotype introgression through functional markers. Previously, selection pressure was observed on *TaSnRK2*.*3*-1A/B (Miao et al., 2017), *TaSnRK2*.*9*-5A (Ur Rehman et al., 2019), *TaSAP*-7B (Wang et al., 2018), *TaMOC*-7A (Zhang B. et al., 2015), *TaLTPs*-1A (Li Q. et al., 2016), *TaPARG*-2A (Li B. et al., 2016), and *TaPPH*-7A (Wang et al., 2019) favorable allelic variations in Chinese wheat cultivars where the frequencies of favored haplotypes had gradually increased from the beginning of the last century.

Both PIC and *H*_*e*_ values were higher in tetraploid wheat as compared to Pakistani hexaploid wheat. Lower PIC and *H*_*e*_ values in Pakistani hexaploid wheat concluded a severe domestication genetic bottleneck. The probable reason for bottlenecking is the genetic structure mainly built around relatively few cultivars such as Bluebird, Kalyansona, Kauz, and Buho, causing serious threat by narrowing the genetic base for drought tolerance-conferring genes. Hence, introgression from geographically different wheat may be a preferred strategy to introduce novel allelic variations at loci conferring drought tolerance for sustainable production.

Finally, our work included a set of genes conferring drought tolerance. We developed a breeding toolkit for high-throughput and cost-effective genotyping for drought-conferring genes in wheat. We believe that this toolkit can accelerate breeding efforts to select diverse and pyramid favorable allelic variations in wheat breeding programs in Pakistan.

## Data Availability Statement

The datasets presented in this study can be found in online repositories. The names of the repository/repositories and accession number(s) can be found in the article/Supplementary Material.

## Author Contributions

SU was responsible for conceptualization. SU, MA, MSd, MK, MN, RI, and AI performed the experiments and analyzed the data. SU wrote the manuscript. ZA, XM, MSj, and RJ reviewed the manuscript and assisted in the completion of the experiments. All authors contributed to the article and approved the submitted version.

## Conflict of Interest

The authors declare that the research was conducted in the absence of any commercial or financial relationships that could be construed as a potential conflict of interest.
